# Oral vitamin B₁₂ after total gastrectomy for gastric cancer: mapping the evidence

**DOI:** 10.1007/s10120-026-01745-8

**Published:** 2026-06-13

**Authors:** Patryk Rogaczewski, Michał Janiak, Justyna Durślewicz, Krzysztof Koper, Jędrzej Borowczak

**Affiliations:** 1 Department of Oncological Surgery, Faculty of Medicine, Ludwik Rydygier Collegium Medicum in Bydgoszcz, Nicolaus Copernicus University in Torun, Toruń, Poland; 2 Department of Tumor Pathology and Pathomorphology, Prof. Franciszek Łukaszczyk Oncology Centre, Bydgoszcz, Poland; 3 Department of Animal Biotechnology and Genetics, Faculty of Animal Breeding and Biology, Bydgoszcz University of Science and Technology, Bydgoszcz, Poland; 4 Clinical Department of Oncology, Prof. Franciszek Łukaszczyk Oncology Centre, Bydgoszcz, Poland; 5 Faculty of Medicine, Bydgoszcz University of Science and Technology, Bydgoszcz, Poland

**Keywords:** Gastrectomy, Vitamin B_12_, Deficiency, Supplementation, Treatment, Review

## Abstract

**Background:**

Patients who undergo total gastrectomy lose the ability to produce intrinsic factor and require lifelong supplementation to prevent vitamin B_12_ deficiency. While oral B_12_ has emerged as a potential alternative to parenteral therapy, data remain limited, and most clinicians still favor intramuscular injections. This scoping review aimed to map the current evidence on the efficacy, safety, and implementation of oral vitamin B_12_ supplementation in patients after total gastrectomy for gastric cancer.

**Methods:**

We included peer-reviewed studies reporting outcomes of oral vitamin B_12_ supplementation after total gastrectomy. A systematic literature search was conducted in scientific databases, including articles published up to November 2025. The reference lists of included articles were screened manually. Extracted data were synthesized narratively. The review was conducted in accordance with the PRISMA-ScR guidelines.

**Results:**

Seven studies (*n* = 449, including 310 patients receiving oral B₁₂) were included. Most used mecobalamin at daily doses of 500–1500 µg. Oral B_12_ consistently normalized serum levels and alleviated symptoms in the majority of patients. Comparative studies showed that, in terms of biochemical response, oral supplementation was not inferior to parenteral therapy. However, symptom monitoring was inconsistent, follow-up durations were generally short (< 6 months), and only one study was randomized. Standardized clinical outcome measures were rarely used, and data from Western populations were limited. Heterogeneity in dosing regimens and B_12_ formulations further limited comparability across studies.

**Conclusions:**

Oral B_12_ appears to be a feasible route of supplementation after total gastrectomy, but current evidence is limited by heterogeneity, underreporting of clinical outcomes, and lack of long-term follow-up. High-quality trials with standardized outcome measures are warranted.

## Introduction

Vitamin B_12_ (cobalamin) is a water-soluble vitamin that plays an important role in the synthesis of DNA and red blood cells, and regulates neural functions [1]. It is synthesized exclusively by microorganisms and must be obtained from dietary sources, primarily animal-derived products. In healthy patients, ingested vitamin B_12_ undergoes a complex process of absorption, which takes place in various parts of the intestinal tract [2]. Initially, vitamin B_12_ binds to haptocorrin, a binding protein secreted by the salivary glands. Upon reaching the small intestine, the B_12_-haptocorrin complex is degraded by pancreatic enzymes, allowing vitamin B_12_ to associate with intrinsic factor (Castle factor), secreted by stomach parietal cells. After being absorbed by the mucosa of the distant ileum via receptor-mediated endocytosis, cobalamin appears in the portal circulation complex with transcobalamin II [3, 4]. Dysregulation or dysfunction of any component involved in this process may impair vitamin B_12_ absorption, resulting in deficiency and associated clinical symptoms.

Stomach cancer is one of the most common malignancies, with over one million new cases diagnosed annually. A substantial proportion of these are detected at an advanced stage due to the disease’s asymptomatic nature in its early phases [5]. The prognosis remains poor, with only 30% of patients surviving 5 year after diagnosis. If feasible, patients usually undergo surgery, with total gastrectomy (TG) being indicated for those with stage IB-III disease [6]. Depending on tumor location and extent, distal, subtotal, or proximal gastrectomy may also be performed; however, only TG eliminates intrinsic factor production and thus uniquely predisposes patients to vitamin B_12_ deficiency. While potentially curative, TG is associated with reduced quality of life and multiple health-related consequences. One of them is the loss of intrinsic factor production, which leads to vitamin B_12_ deficiency and subsequent megaloblastic anemia unless the demand for B_12_ is adequately addressed through supplementation [7, 8].

Current guidelines recommend monitoring serum vitamin B_12_ levels at least every six months in patients after gastrectomy, following the local practice for route of administration [9]. The frequency and dosage should be individually adjusted to maintain concentrations within the reference range [10]. Clinical practice regarding vitamin B_12_ supplementation after gastrectomy also varies internationally, particularly in terms of preferred routes of administration and monitoring intervals. While there are no universal recommendations, most clinical protocols favour intramuscular administration, typically starting with 1000 µg weekly for 5 weeks, followed by monthly injections for a total of three months, and subsequent monitoring every three months [11, 12]. Although intramuscular supplementation remains the most widely used approach, alternative routes have been explored. Oral vitamin B_12_ supplementation has emerged as a potentially effective option, particularly for patients who cannot tolerate or access intramuscular injections. Preliminary data indicate that, despite the lack of intrinsic factor, passive absorption of high-dose B_12_ may be sufficient to maintain adequate serum levels. However, significant gaps remain in the literature regarding the long-term efficacy and safety of oral supplementation after total gastrectomy. Further research is therefore needed to confirm its viability as an alternative to the intramuscular route and to establish evidence-based guidelines for post-gastrectomy care [13].

In this scoping review, we aimed to identify and characterize existing studies evaluating oral vitamin B_12_ supplementation after total gastrectomy, with particular attention to dosing regimens, clinical and biochemical outcomes, and follow-up duration. By synthesizing the available data, we sought to determine whether oral cobalamin may be considered a reliable, evidence-based alternative to parenteral administration in clinical practice.

## Materials and methods

### Protocol and registration

This scoping review was conducted in accordance with the PRISMA-ScR (Preferred Reporting Items for Systematic Reviews and Meta-Analyses extension for Scoping Reviews) guidelines [14]. The protocol was not prospectively registered.

### Eligibility criteria

The main inclusion criterion was the evaluation of oral vitamin B_12_ supplementation in adult patients after total gastrectomy, regardless of the comparator or clinical context. Studies were included if they reported outcomes related to the effectiveness, biochemical response, or clinical consequences of oral B_12_ administration.

Articles were excluded if they focused exclusively on subtotal gastrectomy, gastric bypass, involved only parenteral supplementation, or lacked the assessment of oral B_12_ administration as a separate intervention.

### Search methodology

A literature search was conducted on November 24, 2025, and included studies published in English or Polish up to November 24, 2025. The following databases were searched: PubMed, Cochrane Library, Embase, ClinicalTrials.gov and International Clinical Trials Registry Platform (ICTRP). The search strategy included the keywords: (((B_12_) or (cobalamin) or (mecobalamin) or (methylcobalamin)) and ((gastrectomy) or (total gastrectomy))). Although route-specific terms (e.g. “oral”, “enteral”) were not included in the primary search string, all identified records were manually screened to identify studies reporting outcomes of oral vitamin B_12_ supplementation.

### Study selection

Articles were retrieved and assessed against the eligibility criteria. Study selection was performed by two independent researchers through title and abstract screening, followed by full-text assessment according to predefined inclusion and exclusion criteria. In case of discrepancies, a consensus was made whether an article should be included.

### Data charting and extraction

For each included study, the following data were extracted: author, year, study design, number of patients receiving oral vitamin B_12_, form and dose of supplementation, duration of follow-up, main clinical and biochemical outcomes, and relevant methodological comments. Data were collected independently by two reviewers, charted using Microsoft Excel (version 2021), verified manually against full-text sources, and summarized in Table 1 for comparison and synthesis.

### Critical appraisal

In accordance with the PRISMA-ScR guidelines, no formal risk-of-bias or quality assessment tools were applied. Although no formal risk-of-bias tools were applied, a structured narrative critical appraisal was conducted to support interpretation of the evidence. This appraisal included an assessment of key methodological limitations across all included studies, such as predominantly non-randomized or retrospective designs, small sample sizes, lack of standardized outcome measures, heterogeneous definitions of vitamin B_12_ deficiency, and substantial variation in dosing regimens and supplementation protocols. Importantly, nearly all studies were conducted in East Asian settings, where clinical follow-up structures, dietary patterns, and post-gastrectomy care pathways differ from Western practice, further limiting external validity and generalizability. Reporting quality was inconsistent, with frequent omission of symptom assessments, quality-of-life measures, adherence data, and detailed information on the exact form of oral B₁₂ administered. Follow-up durations were generally short, with only two studies providing ≥ 12 months of observation. These methodological and contextual limitations introduce notable risks of bias and restrict the strength and applicability of available evidence, and were therefore considered when synthesizing and interpreting the results of this scoping review. Key methodological limitations of the included studies were summarized descriptively in Table 1.

### Data synthesis

Given the heterogeneity of study designs, patient populations, and outcomes, no quantitative synthesis or meta-analysis was performed. Instead, a narrative synthesis was conducted. The main study characteristics, interventions, and outcomes were summarized in a structured comparison table (see Table 1). Recurring themes and findings were identified, and methodological limitations were discussed to support interpretation. Evidence was mapped to highlight areas of consensus and gaps in the current literature, informing directions for the future.

## Results

### Selection of sources of evidence

**Fig. 1:**
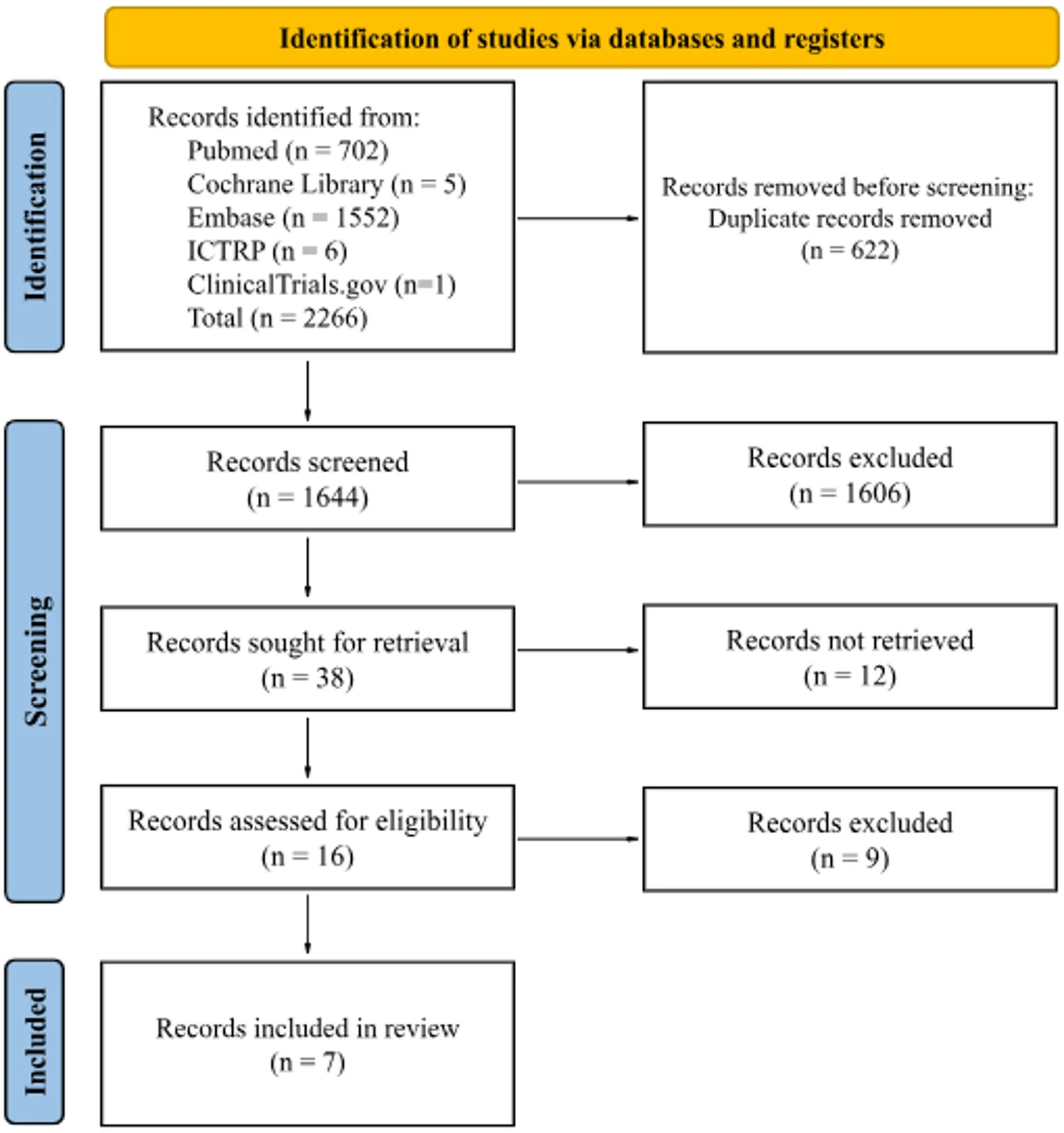
PRISMA-ScR flow diagram showing the study selection process

The initial search yielded 2266 records. After removal of duplicates, 1664 titles and abstracts were screened. Of these, 16 full-text articles were assessed for eligibility. The main reasons for exclusion at full-text stage were: focus on subtotal gastrectomy, exclusive use of parenteral supplementation, and lack of clinical outcomes. Studies that did not allow for separate analysis for patients taking oral B_12_ supplementation were also excluded. The search identified 7 studies that met the eligibility criteria and were included in the analysis. The selection process is illustrated in Fig. 1 (PRISMA-ScR flow diagram).

### Characteristics of sources of evidence

Seven studies were included in the review: three retrospective controlled studies, one randomized controlled trial, one prospective controlled study, one prospective observational study, and one retrospective case series, conducted between 2000 and 2025. Sample sizes ranged from 26 to 133 patients, with the study by Aoyama et al. [15] including the highest number of participants receiving oral vitamin B_12_ (*n* = 74). Most studies administered mecobalamin daily in doses ranging from 500 µg to 1500 µg, while in the studies by Moleiro et al. and Pimiento the type of cobalamin was not specified [16, 17]. Follow-up duration varied from 3 months to 36 months, with only three studies providing data beyond 12 months. Outcomes were primarily biochemical (serum vitamin B_12_ levels); however, several studies also assessed hematologic parameters and clinical symptom resolution. Table 1 summarizes the key characteristics of the included studies.

**Table 1:** Key characteristics of studies on oral vitamin B_12_ supplementation after total gastrectomy

Authors	Study type	*N*	B_12_ oral dosing	Observation time	Results	Commentary*
Adachi et al. [18]	RCS	31 (18 p.o.)	500–1500 µg/day mecobalamin	6 months	Normalization of serum B_12_ levels in all 18 patients receiving oral B_12_; symptom resolution observed; effect independent of dose (500–1500 µg).	Small retrospective series; no standardized outcome measures.
Kim et al. [19]	PC	60 (30 p.o.)	1500 µg/day mecobalamin	3 months	After 30 days of treatment, B_12_ levels increased and were maintained after 90 days; no adverse events reported.	I.m. B_12_ in the control group; not randomized; small sample; follow-up limited to 3 months of treatment.
Rino et al. [11]	RC	73 (73 p.o.)	500–1500 µg/day mecobalamin	Not specified; monitoring every 1–6 months	B_12_ normalized in 85.7% of the oral group; no significant difference vs. i.m.; In 4 (5.5%) patients B_12_ levels decreased despite oral supplementation.	Outcome definition based on lab values only; high baseline B_12_ variability, B_12_ deficiency defined as < 180 pg/ml; finally, all patients switched to oral B_12_.
Moleiro et al. [16]	PO	26 (26 p.o.)	1000 µg/day cobalamin (form unspecified)	8.5–28 months	At 6–24 months, all patients maintained normal B_12_ levels without need for i.m. injections; significant increase in B_12_; no adverse events.	Long follow-up; no control group; all patients switched from i.m. to p.o., limiting causality inference.
Namikawa et al. [20]	RC	133 (46 p.o.)	500 µg/day mecobalamin	3, 6, and 12 months	At 3, 6, and 12 months, B_12_ increased significantly in the oral group; effect equal or superior to i.m.; RBC increased; no symptoms reported.	Retrospective design; unclear adherence; p.o. group relatively small; no symptom-specific outcomes.
Aoyama et al. [15]	RCT	74 (74 p.o.)	500–1500 µg/day methylcobalamin	3 months	B_12_ normalized in 91.7% (500 µg) and 100% (1500 µg); no difference in symptom improvement between both groups; 2 adverse events in the 500 µg group only.	Well-designed RCT; the primary endpoint was the incidence of a normal serum B_12_ level at three months after treatment; short follow-up time; limited power for clinical endpoints. The study was prematurely closed due to poor accrual after reaching 50% of its goal.
Pimiento [17]	RC	52 (43 p.o.)	Cobalamin; dose and form not specified	6, 12, 24, or 36 months	At 6, 12, and 24 months, mean B_12_ ~1000 pg/mL; all patients maintained normal B_12_ levels; 50% of patients remained on oral B_12_ for up to 3 years; Hb and MCV remained normal; no adverse events.	Retrospective study; no standardized symptom evaluation; B_12_ form not specified; while oral B_12_ remained the most common form of supplementation (*n* = 8/16), some patients were transitioned to other forms of B_12_ by their local providers.

*RCS* Retrospective case series; *PC* Prospective controlled; *PO* Prospective observational; *RC* Retrospective cohort; *RCT* Randomized controlled trial; *p.o.* per os; *i.m.* intramuscular

*If not specified otherwise, B_12_ deficiency was defined as vitamin B_12_ levels < 200 pg/mL

### Results of individual sources of evidence

Adachi et al. conducted a retrospective cohort study involving 31 patients with B_12_ deficiency, who received treatment upon diagnosis. The cohort included 23 men and 8 women, with average age of 57.2 years. Of them, 18 received daily oral mecobalamin in doses ranging from 500 to 1500 µg, while the remaining were treated with intramuscular cyanocobalamin. At the start of the treatment, 78% (24/31) of patients reported deficiency-related symptoms, including fatigue (39%), cold feet or legs (32%), and numbness or dizziness (13% each). Serum B_12_ levels increased significantly in both groups. No substantial differences were observed between treatment routes. The authors concluded that enteral vitamin B_12_ supplementation should be prescribed routinely after total gastrectomy, as it rapidly increases serum B_12_ levels and resolves deficiency-related symptoms [18].

Kim et al. [19] conducted an open-label, single-center, prospective controlled study comparing the efficacy and safety of 1500 µg of oral mecobalamin daily for 3 months with a standard intramuscular protocol in groups with 30 patients with B_12_ deficiency each, irrespective of deficiency-related symptoms. In all patients, serum B_12_ levels normalized by day 90. In the oral group, the authors observed a continued increase of serum B_12_ levels, while in the intramuscular group, B_12_ concentration peaked after the weekly boosting injection period, decreased at 2 months and stabilized at day 90. In both groups, homocysteine concentrations decreased, and no adverse effects were reported. Interestingly, in the oral B_12_ group, serum folate level decreased but remained in the normal reference range. At the start of the treatment, 29 of 30 patients had deficiency-related symptoms, all of which resolved after 1 month of oral vitamin B_12_ replacement. The authors concluded that oral vitamin B_12_ replacement is an effective and safe treatment for vitamin B_12_ deficiency after total gastrectomy.

In a retrospective cohort study, Rino et al. [11] evaluated various routes of vitamin B_12_ supplementation in 73 patients after total gastrectomy. Among them, 28 received daily oral mecobalamin (500–1500 µg), 42 received intramuscular cyanocobalamin (500 µg) every 1–3 months, and 3 received intravenous cyanocobalamin (500 µg) every 1–3 months. Serum vitamin B_12_ levels normalized in 85.7% (24/28) of patients in the oral group, compared to 76.2% (32/42) in the intramuscular group and 66.7% (2/3) in the intravenous group, with no statistically significant differences between administration routes. After switching all patients to oral supplementation, B_12_ levels remained subnormal in only 4 patients (5.5%) and subsequently normalized in all after continued therapy. The authors concluded that daily oral vitamin B_12_ supplementation, typically at 500 µg, may be sufficient and effective for preventing deficiency after total gastrectomy, although they emphasized that replacement therapy is necessary and should be continued long term.

The prospective observational study by Moleiro et al. [16] followed 26 patients, aged 29–79, who received 1000 µg of oral cobalamin daily. At baseline, all patients had normal B_12_ levels, 17 had previously received intramuscular B_12_, while 9 had not started treatment. After switching to oral supplementation, B_12_ levels remained within the reference range throughout the 20 month follow-up in all patients, with no need for intramuscular supplementation. Interestingly, B_12_ levels increased steadily during the first 12 months and stabilized thereafter. No patient experienced deficiency-related symptoms or supplementation-related adverse events. The authors concluded that oral supplementation is effective and safe after total gastrectomy and should be considered the preferred route in long-term care.

Namikawa et al. [20] retrospective study included 133 Japanese post-gastrectomy patients who underwent Roux-en-Y or double tract reconstruction. B_12_ deficiency was present in 71.4% (95/133), among whom 46 received oral mecobalamin (500 µg) daily and 23 were supplemented with intramuscular mecobalamin (500 µg) every 3 months. After supplementation, serum B_12_ levels increased in all patients, irrespective of the administration route. At 3, 6, and 12 months, B_12_ levels were significantly higher in the oral group than in the intramuscular group. The authors reported significant improvement in anemia parameters following the start of oral B_12_ and noted no significant differences regarding the red blood cell count, hematocrit and hemoglobin concentration between the two group. The authors concluded that enteral vitamin B_12_ supplementation might be effective in improving anemia in this patient group.

Aoyama et al. [15] conducted the only randomized, open-label, multicenter, controlled phase II study comparing the efficacy of 500 µg versus 1500 µg of oral methylcobalamin daily in 74 patients with B_12_ deficiency after total gastrectomy. Of them, 36 were assigned to the 500 µg/day group and 38 to the 1500 µg/day group. After 3 months, serum B_12_ levels increased in all patients and reached normal levels in 91.7% of patients in the 500 µg group and in 100% of those receiving 1500 µg. Baseline clinical symptoms included primarily tiredness, memory problems, and tingling sensation in the hands or feet. The frequency of deficiency-related symptoms decreased following the supplementation, with no statistically significant differences between groups. Two grade 2 adverse events were reported in the lower-dose group (ileus and anemia), while none occurred in the high-dose group. The authors concluded that, although the primary endpoint was not met, oral vitamin B_12_ at 500 µg/day appeared as effective and safe as the 1500 µg/day regimen, and noted that the study was prematurely closed due to poor accrual.

In a recent retrospective analysis, Pimiento assessed the efficacy of oral cobalamin 52 patients who underwent total gastrectomy. Initially, oral supplementation alone was started in 43 patients, while two received a combination of oral and intramuscular supplementation, six received intramuscular B_12_, and one preferred subcutaneous injections. The specific dosages used for oral and parenteral supplementation were not reported. All patients maintained normal or above-normal serum B_12_ levels, with minimal fluctuation observed between follow-up visits. While oral B_12_ remained the most common form of supplementation (*n* = 8/16), some patients were transitioned to other routes by their local providers. There was also no association between the initial B_12_ administration route, hematocrit, and serum hemoglobin concentration. The authors were unable to report on peripheral neuropathy as a consequence of vitamin deficiency, since most patients received systemic therapy. However, among the nine patients who did not receive chemotherapy, symptoms of peripheral neuropathy did not occur. The authors concluded that oral vitamin B_12_ administration is a safe, feasible, and convenient long-term supplementation route after total gastrectomy, while emphasizing the need for continued monitoring of B_12_ levels [17].

### Synthesis of results

Despite notable heterogeneity in study design, population size, and follow-up duration, the studies consistently reported biochemical improvement with oral vitamin B_12_ supplementation as feasible long-term approach for preventing and managing post-gastrectomy vitamin B_12_ deficiency (Table 1). Across all seven studies, daily administration of mecobalamin in doses ranging from 500 to 1500 µg consistently led to normalization of serum B_12_ levels and resolution of clinical symptoms in the vast majority of patients. Early evidence from Adachi and Kim challenged the long-standing view that only parenteral routes were effective, demonstrating that enteral absorption can be sufficient even in the absence of intrinsic factor. Subsequent prospective data (e.g., Moleiro) and long-term retrospective analyses (e.g., Pimiento) confirmed these results in more heterogenous populations. The only randomized study to compare 500 µg versus 1500 µg daily [15] demonstrated a dose-dependent trend favoring 1500 µg, although the difference was not statistically significant. Although 500 µg/day was effective in most cases, the optimal dose remains to be defined in adequately powered trials.

Serum B_12_ levels generally normalized within 3 months, and adherence, rather than dose, emerged as the primary determinant of success. In comparative studies, oral supplementation was at least non-inferior to parenteral administration, with higher mean B_12_ concentrations reported in the oral arm (Namikawa et al.). There were no statistically significant differences in biochemical response (e.g. Adachi et al., Kim et al., Rino et al.).

Across the included studies, biochemical deficiency did not always correlate with symptom burden. This was particularly evident in studies by Adachi et al., Kim et al., and Aoyama et al., where baseline symptom burden did not consistently align with serum B_12_ concentrations. Several patients remained asymptomatic despite low serum B_12_ levels, whereas others reported fatigue, paresthesia, or cognitive complaints despite biochemical normalization. This inconsistency reflects the well-described clinical-biochemical gap in vitamin B_12_ monitoring and underscores the need for standardized assessment of both laboratory and symptom-based outcomes. However, clinical outcomes were reported heterogeneously and often descriptively, which limits direct comparison across studies.

Although symptom reporting was inconsistent across studies, improvement was observed when formally assessed. In studies by Adachi, Kim, and Aoyama, fatigue, paresthesia, or cognitive complaints improved following oral therapy, with full resolution in most cases. Hematologic parameters also improved in studies that included such endpoints, particularly in Namikawa et al., where oral B_12_ was associated with normalization of anemia markers. Importantly, the therapy was well tolerated and no related serious adverse events were reported; only two adverse events occurred in the Aoyama et al. study.

Follow-up duration varied from 3 to 36 months, with sustained normalization of serum B_12_ observed in long-term studies (e.g., Moleiro, Pimiento). However, several studies lacked detailed symptom tracking, quality-of-life data, or standardized definitions of response. Additionally, the specific form and dose of oral B_12_ were not always reported, limiting interpretability. Although the included studies consistently reported biochemical improvement with high-dose oral vitamin B_12_ supplementation after total gastrectomy, the certainty of this evidence is limited. These findings should be interpreted with caution given the methodological constraints of the available studies, including small sample sizes, short follow-up, inconsistent reporting of clinical endpoints, and the predominance of East Asian populations. Further well-designed, long-term studies are required to clarify the role of oral supplementation in routine practice.

## Discussion

Under normal physiological conditions, vitamin B_12_ absorption relies on a series of carrier proteins and intrinsic factor, enabling active uptake in the terminal ileum [21]. The absence of intrinsic factor following total gastrectomy shuts down the active absorption of vitamin B_12_ from the gastrointestinal tract, leaving passive diffusion as the only mechanism of B_12_ absorption. Without adequate supplementation, clinical symptoms of B_12_ deficiency typically emerge within a year, reflecting the gradual depletion of hepatic vitamin B_12_ stores [20].

Passive absorption is fast, but inefficient. Although only ~ 1% of an oral dose is absorbed via passive diffusion, this is sufficient to maintain adequate serum concentrations and prevent vitamin B_12_ deficiency when high daily doses of vitamin B_12_ are administered [22]. If the oral dose of B_12_ falls within the range of 500–1500 µg per day, then, assuming the absorption of ~ 1%, this translates to 5–15 µg of vitamin B_12_ entering the bloodstream daily. This exceeds the recommended daily requirement of 2.4 µg for adults, and provides the mechanistic rationale on why oral doses of B_12_ are effective even in the absence of intrinsic factor [11, 18, 23].

The evaluation of clinical symptoms of vitamin B_12_ deficiency following total gastrectomy was significantly limited in most of the analyzed studies. Only a few trials monitored the presence or resolution of neurological, hematological, or general symptoms. The lack of methodological standardization precluded meaningful comparisons across studies. None of the included works employed validated symptom assessment tools or clearly defined criteria for clinical improvement.

Most included studies originated from East Asia, where several aspects of perioperative practice differ from those in Western countries. This includes more frequent D2 lymphadenectomy in high-volume East Asia centers and differences in reconstruction techniques [24]. Postoperative surveillance is also less standardized in Western settings and is often symptom-driven, in contrast to more structured schedules used in East Asia [25, 26]. Monitoring and supplementation practices for B_12_ vary as well, with Western recommendations allowing either oral or parenteral replacement, while Asian guidelines tend to prefer high-dose oral regimens [25]. These regional differences may affect biochemical monitoring, symptom detection, and timing of supplementation, thereby limiting the generalizability of the East Asian findings to other populations.

An important finding of this review is the persistent clinical-biochemical gap observed across studies. Serum vitamin B_12_ is an imperfect surrogate for functional status, and deficiency may not manifest clinically until tissue stores are significantly depleted [27]. Conversely, nonspecific symptoms such as fatigue, neuropathic complaints, or cognitive slowing may overlap with postoperative complications, anemia of mixed etiology, or nutritional deficiencies unrelated to B_12_. Moreover, serum B_12_ concentrations do not capture the bioactive fraction of the vitamin, and markers such as methylmalonic acid or homocysteine were rarely assessed in the included studies [28]. As a result, several patients reported symptoms despite “normal” B_12_ levels (e.g., Aoyama et al. and Kim et al.), whereas others remained asymptomatic despite biochemical deficiency (e.g., Adachi et al., Park et al.). This discrepancy underscores the need for combined biochemical and clinical monitoring and highlights that reliance on serum B_12_ alone may lead to under- or overtreatment in routine practice.

In the study by Kim et al. [19] after three months of oral supplementation, 28 out of 29 patients reported symptom improvement, and 16 experienced complete resolution. However, the assessment was entirely subjective and lacked formal quantification. Similarly, Aoyama et al. [15] observed symptom relief in both oral methylcobalamin dosing groups (500 µg/day and 1,500 µg/day), but the evaluation relied on patient-reported outcomes without detailed quantitative or statistical analysis of symptom severity.

Furthermore, the disconnect between laboratory normalization and persistent clinical complaints raises concerns about relying solely on serum vitamin B_12_ levels as an indicator of therapeutic success. Some patients continue to report fatigue, paresthesias, or cognitive disturbances even after biochemical parameters return to normal, suggesting that symptom resolution may lag behind or occur independently of serum correction [19]. This observation emphasizes the importance of incorporating structured neurological and functional assessments into follow-up protocols. Standard laboratory markers, while necessary, may not fully reflect the neurophysiological recovery process, especially in patients with preexisting or prolonged deficiencies.

Another major limitation observed across the studies was the short duration of follow-up. Most trials assessed the efficacy of oral vitamin B_12_ supplementation over a period of only 3 to 6 months, precluding robust conclusions regarding long-term therapeutic durability and the prevention of delayed complications [15, 19]. Only a few studies, such as that by Moleiro et al. and Pimiento, extended follow-up beyond 24 months; however, even in those cases, clinical symptom monitoring was either non-standardized or limited to general subjective reporting [16].

Given the gradual nature of post-gastrectomy B_12_ depletion, often developing over 12 to 36 months, short-term studies fail to capture the full spectrum of clinical outcomes [16]. Future trials should therefore implement longer follow-up periods, ideally spanning several years, and employ validated tools for symptom tracking and neurocognitive performance. The current body of evidence underscores the urgent need for prospective studies with standardized and objective assessment of clinical symptoms, accompanied by long-term follow-up. Such studies are essential to evaluate not only biochemical efficacy but also the true impact of oral vitamin B_12_ supplementation on neurological function and quality of life in patients after total gastrectomy. Additionally, stratifying outcomes by baseline deficiency severity, comorbidities, and duration of prior parenteral therapy may help identify patient subgroups at higher risk of incomplete recovery despite oral supplementation. Addressing these methodological gaps is crucial for determining whether oral cobalamin can be considered not only biochemically effective, but clinically equivalent to parenteral administration in the long-term care of post-gastrectomy patients.

In addition to clinical outcomes, the route of vitamin B_12_ administration may improve patient experience and healthcare efficiency. Studies suggest that most patients prefer oral medications over intramuscular injections, primarily due to convenience [29, 30]. Therefore, switching to oral supplementation would not only improve the patient’s perception of the received treatment, but also increase adherence by minimizing barriers such as limited mobility, side effects of intramuscular injections, pain, or logistical burdens. A more positive treatment experience introduces a psychological factor that may influence patient attitude and quality of life, potentially contributing to improved outcomes [31]. From a healthcare system perspective, shifting to home-based oral therapy has the potential to reduce outpatient workload, lower treatment-related costs, and optimize resource use, especially at tertiary cancer centers. While patient preference and quality of life were not formally assessed in the reviewed studies, these aspects warrant closer attention in future research to better reflect real-world treatment benefits.

Most studies included in this review used mecobalamin, the coenzyme form of vitamin B_12_ [19, 15, 11], which may offer improved tissue uptake compared to cyanocobalamin, although direct comparative data are lacking. Recent studies suggest that supplementation with natural B_12_ forms, including methylcobalamin, is preferred over cyanocobalamin due to their greater bioavailability and improved safety profile [32]. However, the specifics of the target population, including the loss of intrinsic factor-mediated absorption and higher cost of methylcobalamin compared to cyanocobalamin, may guide treatment choices. Nevertheless, given that hepatic B_12_ stores typically suffice for 9–12 months, the delayed onset of deficiency symptoms highlights the importance of early and sustained oral replacement [18, 20].

## Limitations

This scoping review comes with several limitations related to the design and quality of the included studies, as well as the availability of guideline-based data. First, the number of studies investigating the efficacy of oral vitamin B_12_ supplementation after total gastrectomy remains limited, as shown in the PRISMA-ScR flow diagram (Fig. 1). Consequently, the available data, outcomes and conclusions are based on a relatively small population of 449 patients, of whom 310 received oral therapy. Second, follow-up duration was generally short and insufficient to draw conclusions regarding the long-term biochemical stability of vitamin B_12_ levels and the resolution of clinical symptoms (Table 1). Only one of the seven included studies was a randomized controlled trial, with a follow-up time of just three months. Additionally, symptoms assessment across studies was heterogenous and largely subjective. None of the reports used standardized clinical tools or functional markers such as methylmalonic acid or homocysteine, which further limits the interpretability of clinical-biochemical correlations. Moreover, the predominance of East Asian studies, where surgical techniques, follow-up schedules, and vitamin B_12_ supplementation practices differ from Western settings, further limits the generalizability of our findings. Finally, since current guidelines recommend parenteral B_12_ after gastrectomy, structured data on oral supplementation are still limited. Most reports reflect individual clinical decisions rather than standardized institutional protocols - which, ironically, is part of the reason we need a review like this in the first place.

## Conclusions

All included studies reported that oral B_12_ supplementation was biochemically effective and well tolerated after total gastrectomy, although the strength of evidence remains limited by small sample sizes, heterogenous outcome reporting, and predominantly short follow-up durations. While available data suggest that oral therapy can achieve target serum B_12_ levels in most patients, the clinical relevance of these findings is difficult to fully assess given the inconsistent evaluation of symptoms and the lack of functional biomarkers in the included studies.

Future research should prioritize long-term, prospective designs incorporating standardized definitions of biochemical response, validated clinical assessments, and functional markers such as methylmalonic acid or homocysteine. Randomized trials comparing different oral dosing strategies and formulations would be particularly valuable, especially in non-Asian populations where practice patterns may differ. To support such efforts, we summarized the major evidence gaps identified in this review in Table 2.

Although patient preference and the potential logistical advantages of oral therapy were not systematically assessed in the available literature, these aspects deserve greater attention in future studies to better reflect real-world care models. Until higher-quality evidence becomes available, decisions regarding route of supplementation and follow-up frequency should be individualized based on patient characteristics, local practice, and biochemical monitoring.

**Table 2:** Evidence map for included studies and key reporting gaps

Study	Form of B_12_	Comparator	Clinical outcomes	≥ 12 mo follow-up	Symptom assessment
Adachi et al. [18]	Mecobalamin	i.m.	Yes	No	Yes
Kim et al. [19]	Mecobalamin	i.m.	Yes	No	Yes
Rino et al. [11]	Mecobalamin	i.m., i.v.	No	No	No
Moleiro et al. [16]	Unspecified	–	Yes	Yes	Yes*
Namikawa et al. [20]	Mecobalamin	i.m.	Yes	Yes	No
Aoyama et al. [15]	Methylcobalamin	–	Yes	No	Yes
Pimiento [17]	Unspecified	i.m., s.c.	Yes	Yes	No**

*No patients presented symptoms associated to vitamin B_12_ deficiency

**The authors were unable to distinguish whether neuropathy was related to B_12_ deficiency or past systemic therapy

## Data Availability

The data presented in this study are available from the Corresponding Author upon request.
